# The application of simultaneous mainstem bronchus dilation with pulmonary artery stenting in the context of hypoplastic left heart syndrome

**DOI:** 10.1093/jscr/rjad236

**Published:** 2023-05-29

**Authors:** Salman S Hasan, Elena E Skaribas, Elaijah Islam, David Z Allen, Sancak Yuksel

**Affiliations:** Department of Internal Medicine, McGovern Medical School, Houston, TX, USA; McGovern Medical School, University of Texas Health Science Center, Houston, TX, USA; McGovern Medical School, University of Texas Health Science Center, Houston, TX, USA; Department of Otorhinolaryngology–Head and Neck Surgery, McGovern Medical School, Houston, TX, USA; Department of Otorhinolaryngology–Head and Neck Surgery, McGovern Medical School, Houston, TX, USA

**Keywords:** Bronchial dilation, Balloon dilation, Pulmonary artery stenosis, Direct laryngoscopy, Hypoplastic left heart syndrome, Pulmonary artery stenting

## Abstract

Hypoplastic left heart syndrome (HLHS) is a congenital diagnosis that necessitates immediate intervention at the beginning of life to ensure survival past infancy and to optimize left-side cardiac function. Often, these required procedures can lead to deleterious side effects and resultant complications. In this case report, we present a 15-month-old patient with HLHS who underwent multiple procedures, including two aortic arch surgeries. After the interventions, the patient experienced left main pulmonary bronchus compression along with pulmonary artery stenosis. In this case, we outline an approach to performing vascular dilation without compromise of airway patency.

## INTRODUCTION

Hypoplastic left heart syndrome (HLHS) is a debilitating, congenital diagnosis that necessitates urgent treatment at the beginning of life [1]. The presentation and pathophysiology are characterized by atresia or stenosis and eventual maldevelopment of the left heart system with coarctation of the aorta [2]. To ensure survival past infancy, multiple subsequent operations must be performed to further optimize left-sided function [1, 3, 4]. Often, these procedures can lead to deleterious side effects and resultant complications. For example, the intrathoracic location of the left pulmonary bronchus places it at risk for compression after procedures involving the aorta [5, 6, 7]. In this case report, we present a patient who underwent multiple procedures, including two aortic arch surgeries. After the interventions, the patient experienced left main pulmonary bronchus compression along with pulmonary artery stenosis, a complication rarely encountered for which treatment has not been described completely within the available literature. Pediatric otolaryngology was consulted to assist with the aberrant pulmonary anatomy.

## CASE REPORT

We present a 15-month-old, ex 34-week-old patient with a diagnosis of HLHS with mitral atresia, aortic atresia and coarctation of the aorta. After birth he needed bilateral pulmonary artery banding with subsequent Norwood and Sano procedures. His postoperative course was complicated by tricuspid regurgitation and aortic arch obstruction, which necessitated ascending aortic and aortic arch augmentation along with tricuspid valve repair at the time of the bidirectional Glenn procedure. He ultimately needed a second ascending aorta and aortic arch augmentation and open hybrid standing of the proximal descending aorta with a stent. After these procedures, he was noted to have a narrowing of his left mainstem bronchus and pulmonary artery. Pediatric otolaryngology was asked to assist with left pulmonary bronchus dilation in conjunction with pulmonary artery stent placement.

**Figure 1:**
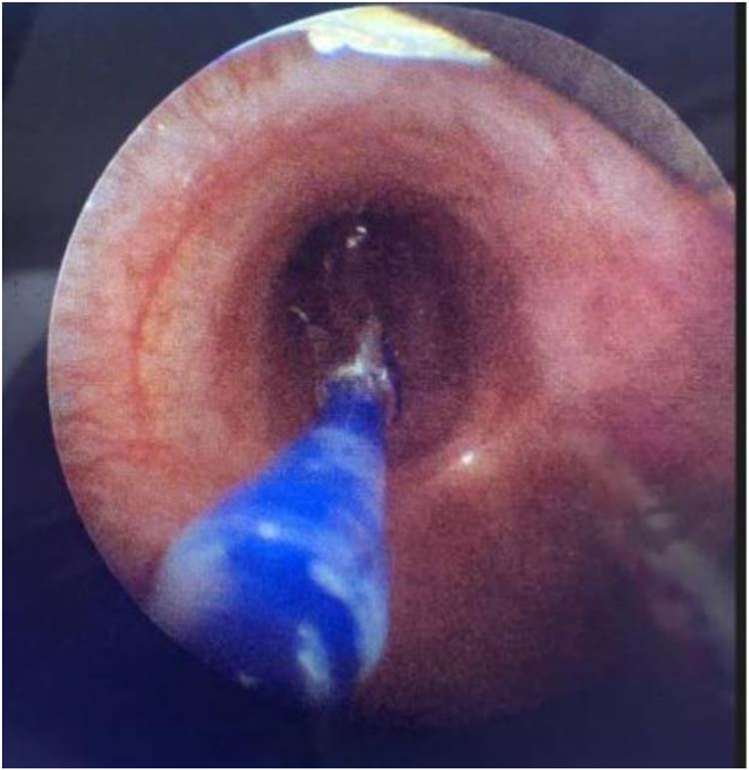
Direct visualization of a 5 mm balloon over a 3.5 mm bronchoscope dilating the left main bronchus.

**Figure 2:**
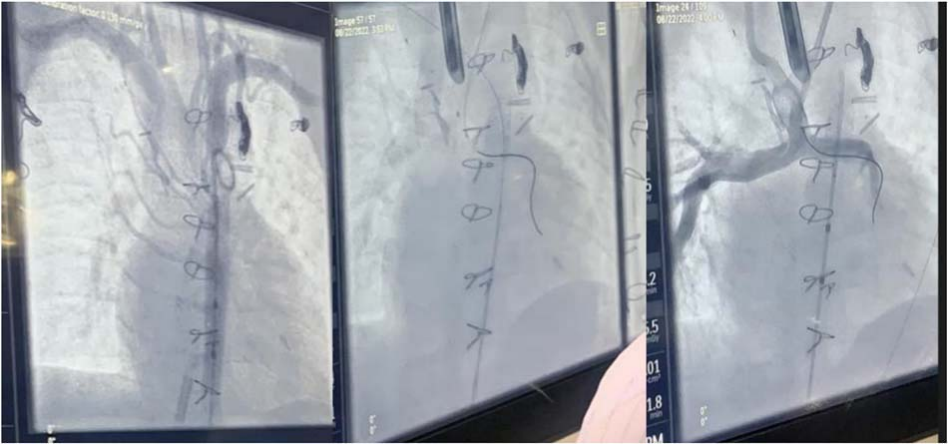
Fluoroscopic intraoperative images of left mainstem bronchus balloon dilation.

During the procedure in the catheter lab, our team placed a 5 mm balloon over a 3.5 mm bronchoscope, shown in Fig. 1, through the suction port and advanced to the level of the carina. Utilizing fluoroscopy, the balloon was advanced to the narrowed portion of the left main stem bronchus. After this placement was confirmed, the interventional cardiology team tunneled their balloons into the left pulmonary artery and aorta. Markers on the balloons were utilized to ensure the airway and intravascular balloons were adjacent. At this time, both intravascular balloons and the bronchial balloon were simultaneously inflated under direct fluoroscopic visualization, shown in Fig. 2. The bronchial balloon was deflated and removed at this time. The left mainstem bronchus was then visualized, whereas the intravascular dilation and stent placement occurred. During this step of the procedure, the left mainstem bronchus appeared wider compared with its preoperative state and no narrowing of the bronchus was observed upon additional intravascular balloon dilation. The patient improved dramatically within the postoperative period, however needed a further cardiac catheterization to dilate his aortic and pulmonary artery stent. Pediatric otolaryngology was again involved to inflate a balloon to prevent further bronchial compression. Once appropriately prepped, we introduced a 3 mm bronchoscope to the left main bronchus. Under direction of the interventional cardiologist, the balloon introduced into the left mainstem bronchus was visualized under fluoroscopy, advanced to the appropriate position determined by the interventional cardiologist, and then inflated. The balloon size was 6 mm in diameter and 20 mm in length and it was inflated to atmospheric pressure and kept inflated while the interventional cardiologist performed their balloon dilation of left pulmonary artery along with a stent placed in the left pulmonary artery and aorta. The balloon was then deflated and withdrawn and the resultant bronchus was larger in comparison to pre-op. The patient tolerated the operation well.

## DISCUSSION

Stenosis and compression of the left main bronchus are complications of aortic arch and cardiac procedures, which can lead to airway obstruction and poor respiratory status [6, 7]. Herein, we describe a pediatric patient that had subsequent airway stenosis secondary to direct vascular compression resulting from multiple cardiac procedures. Most commonly, patients with airway narrowing will undergo a dedicated dilation procedure with interventional pulmonology or otolaryngology. However, when the etiology of the bronchial narrowing is secondary to vascular compression, this allows for the possibility of performing a combination procedure to address both issues in one operation. While balloon dilation for airway stenosis is not a novel concept, there are no reports showcasing the efficacy of bronchial dilation with simultaneous stenting of the great vessels. This reported case demonstrates the value of performing both procedures at once. By dilating the airway at the same time as the pulmonary artery and aorta, we were able to visually observe the patency of the bronchus in real time. This real time visualization also proved advantageous in allowing optimal dilation of vascular structures, which previously had not been possible because of concern for further compression of the bronchus without direct view of the airway via bronchoscopy.

## CONCLUSION

We have detailed a successful intervention of concomitant aortic and pulmonary bronchial dilation in an at-risk pediatric patient. Because of the manipulation of the anatomy of the great vessels, patients with HLHS will undoubtedly be predisposed to developing recurrent pulmonary arterial stenosis necessitating further procedures. In this case, we have outlined a possible approach to performing safe vascular dilation without compromise of airway patency. The most obvious benefit of this procedure is reduced airway stenosis, but another advantage is reducing the number of overall procedures. Patients with HLHS are already in a fragile physiological state and avoiding general anesthesia could be considered a beneficial outcome. The simultaneous bronchial and intravascular dilation procedure proves to be an efficacious and reasonable approach in specific patients.

## References

[1] Feinstein JA, Benson DW, Dubin AM, Cohen MS, Maxey DM, Mahle WT, et al. Hypoplastic left heart syndrome: current considerations and expectations. J Am Coll Cardiol 2012;59:S1–42. 22192720 10.1016/j.jacc.2011.09.022 PMC6110391

[2] Noonan JA, Nadas AS. The hypoplastic left heart syndrome; an analysis of 101 cases. Pediatr Clin North Am 1958;4:1029–56. 10.1016/s0031-3955(16)30727-1 13600906

[3] d’Udekem Y, Iyengar AJ, Galati JC, Forsdick V, Weintraub RG, Wheaton GR, et al. Redefining expectations of long-term survival after the Fontan procedure: twenty-five years of follow-up from the entire population of Australia and New Zealand. Circulation 2014;130:S32–8. 25200053 10.1161/CIRCULATIONAHA.113.007764

[4] Norwood WI, Lang P, Hansen DD. Physiologic repair of aortic atresia-hypoplastic left heart syndrome. N Engl J Med 1983;308:23–6. 6847920 10.1056/NEJM198301063080106

[5] Alsoufi B, Mori M, Gillespie S, Schlosser B, Slesnick T, Kogon B, et al. Impact of patient characteristics and anatomy on results of Norwood operation for hypoplastic left heart syndrome. Ann Thorac Surg 2015;100:591–8. 26138769 10.1016/j.athoracsur.2015.03.106

[6] Moszura T, Mazurek-Kula A, Dryzek P, Sysa A. Bronchial compression as adverse effect of left pulmonary artery stenting in a patient with hypoplastic left heart syndrome. Pediatr Cardiol 2010;4:530–3. 10.1007/s00246-009-9601-4 19937008

[7] Ferandos C, El-Said H, Hamzeh R, Moore JW. Adverse impact of vascular stent ‘mass effect’ on airways. Catheter Cardiovasc Interv 2009;74:132–6. 19213071 10.1002/ccd.21945

